# High-Dose Intravenous Immunoglobulin in Skin Autoimmune Disease

**DOI:** 10.3389/fimmu.2019.01090

**Published:** 2019-06-11

**Authors:** Jochen H. O. Hoffmann, Alexander H. Enk

**Affiliations:** Department of Dermatology, University of Heidelberg, Heidelberg, Germany

**Keywords:** scleromyxedema, vasculitis, IVIg, bullous pemhigoid, pemphigus vulgaris, epidermolysis bullosa acquisita, systemic lupus erythematosus, dermatomyositis

## Abstract

The immunomodulatory potential and low incidence of severe side effects of high-dose intravenous immunoglobulin (IVIg) treatment led to its successful application in a variety of dermatological autoimmune diseases over the last two decades. IVIg is usually administered at a dose of 2 g per kg body weight distributed over 2–5 days every 4 weeks. They are most commonly used as a second- or third-line treatment in dermatological autoimmune disease (pemphigus vulgaris, pemphigus foliaceus, bullous pemphigoid, mucous membrane pemphigoid, epidermolysis bullosa acquisita, dermatomyositis, systemic vasculitis, and systemic lupus erythematosus). However, first-line treatment may be warranted in special circumstances like concomitant malignancy, a foudroyant clinical course, and contraindications against alternative treatments. Furthermore, IVIg can be considered first line in scleromyxedema. Production of IVIg for medical use is strictly regulated to ensure a low risk of pathogen transmission and comparable quality of individual batches. More common side effects include nausea, headache, fatigue, and febrile infusion reactions. Serious side effects are rare and include thrombosis and embolism, pulmonary edema, renal failure, aseptic meningitis, and severe anaphylactic reactions. Regarding the mechanism of action, one can discriminate between functions of the Fcγ region and the F(ab)2 region and their effects on a cellular level. These functions are not mutually exclusive, and more than one pathway may contribute to the beneficial effects. Here, we present a historical background, details on manufacturing, hypotheses on the mechanisms of action, information on the clinical application in the abovementioned conditions, and a brief outlook on future directions of IVIg treatment in dermatology.

## Introduction

The immunomodulatory potential and low incidence of severe side effects of high-dose intravenous immunoglobulin (IVIg) treatment led to its successful application in a variety of dermatological autoimmune diseases over the last two decades. From a historical perspective, the first well-documented proof-of-concept medical application of IVIg in an autoimmune disease dates back to 1981, when Imbach et al. (1) successfully treated a child with immune thrombocytopenia (ITP, formerly idiopathic thrombocytopenic purpura), a humoral and cellular autoimmune reaction against thrombocytes and megakaryocytes that results in skin purpura and organ hemorrhage. Only a few years later, Furusho et al. (2) and Newburger et al. (3) reported the successful application of IVIg in Kawasaki disease, an acute, highly febrile necrotizing systemic vasculitis that mainly affects young children, often manifests with a mucocutaneous rash and unilateral cervical lymphadenopathy, and can be complicated by potentially fatal cardiovascular involvement. Even though, to date, no randomized clinical trial exists, IVIg has since become a mainstay in the treatment of Kawasaki disease. As early as 1993, Dalakas et al. (4) reported the first randomized controlled clinical trial of IVIg in a dermatological condition demonstrating its successful application in dermatomyositis. Subsequently, IVIg has evolved as a treatment for several other dermatological autoimmune conditions, which are listed in Table 1. The clinical application of IVIg is, however, limited by its high cost. Therefore, to warrant a rational use of IVIg, a comprehensive clinical guideline for the use of IVIg in dermatology was first published by the European Dermatology Forum in 2009 and was recently updated in its third edition (5).

**Table 1:** Selection of autoimmune diseases with prominent cutaneous involvement successfully treated with IVIg and common treatment regimes.

**Disease entity**	Common treatment regimes^*^
**Autoimmune bullous dermatoses**	
-Pemphigus vulgaris, Pemphigus foliaceus	2 g/kg bw over 2–5 days q4w
-Bullous pemphigoid	“
-Epidermolysis bullosa acquisita	“
-Mucous membrane pemphigoid	“
**Collagen vascular diseases**	
-Systemic lupus erythematosus	0.4–2 g/kg bw over 2–5 days q4w
**Inflammatory myositides**	
-Dermatomyositis	2 g/kg bw over 2–5 days q4w
**Systemic vasculitides**	
-ANCA-associated vasculitis	2 g/kg bw over 2–5 days q4w
-Kawasaki disease	2 g/kg bw over 12 h
**Scleromyxedema**	2 g/kg bw over 2–5 days q4w

q4w, every 4 weeks; bw, body weight; IVIg, intravenous immunoglobulin.

* *IVIg is mostly given as an adjuvant treatment*.

## Manufacturing

Production of IVIg for medical use is strictly regulated to ensure a low risk of pathogen transmission and comparable quality of individual batches. Immunoglobulin concentrations in healthy human sera range from 7 to 12 g/l. Seventy-five percent of these immunoglobulins are class IgG. Modern multistep procedures allow for the purification of up to 4.5 g IgG with more than 95% purity from 1 L of plasma. Special care is taken to reduce the amount of IgA in the preparation, which can trigger severe anaphylactic reactions in patients with selective IgA deficiency. Usually, plasma from whole-blood donations or plasmapheresis of more than 10,000 donors is pooled into a single batch of IVIg. Potential pathogens are mostly inactivated during the purification process. Further steps to ensure a low risk of pathogen transmission include the epidemiological screening of donors and virological testing of donor blood and pooled plasma. Commercial IVIg preparations are usually distributed with a concentration of 50–100 g/l. Aside from IgG, the preparations usually contain sugars (sucrose, maltose, glucose), glycine, sodium chloride, and albumin. While it remains elusive whether commercial preparations differ regarding their immunomodulatory properties, the sugars contained may warrant a different approach depending on comorbidities (e.g., low glucose for diabetic patients, low sucrose for patients with renal failure, low sodium for patients with cardiovascular disease).

## Practical Considerations and Side Effects

IVIg in the treatment of dermatological conditions is usually administered at a dose of 2 g per kg body weight distributed over 2–5 days every 4 weeks. For comparison, this dose is roughly four times higher than the 0.2–0.8 g per kg body weight used in the setting of substitution therapy. Given the half-life of IgG of approximately 3–4 weeks, application of 2 g/kg body weight IVIg over 2–5 days results in high peak serum concentrations. These peaks are considered by some to contribute to the beneficial treatment effects, even though no confirming data exist. At least in the treatment of immune neuropathies, an increasing body of evidence suggests that subcutaneous application of IgG with lower peak concentrations has a similar treatment effect as IVIg (6). The maximum speed of the infusion depends on the individual patient and IVIg preparation. If tolerated, it may reach 0.01 g per kg body weight per minute. Patients should drink sufficient amounts of water before and during the infusion to optimize tolerance. Adequate urine production and blood pressure should be monitored during the infusion.

More common side effects, experienced by up to 10% of patients, include nausea, headache, fatigue, and febrile infusion reactions. Oftentimes, these mild side effects can be controlled by improved hydration; modification of the infusion speed; or anti-allergic, analgesic, and antipyretic co-medication. Hemolysis and neutropenia, which are mostly mild, are thought to occur due to transferred blood-group antibodies and anti-neutrophil antibodies, respectively. Serious side effects are rare and include thrombosis and embolism, pulmonary edema, renal failure, aseptic meningitis, and severe anaphylactic reactions. These latter side effects are, in part, thought to result from impaired rheology, hypervolemia, and allergic reactions to residual IgA in the preparation. The latter reaction is mainly observed in patients with selective IgA deficiency, a rather common [prevalence of ~1:640 in industrialized western countries (7)], and except for an often mild propensity for airway infections, mostly asymptomatic defect of humoral immunity. Surprisingly, IVIg is sometimes tolerated even in this population, rendering the precise role of anti-IgA antibodies and cofactors like the route of administration in causing allergic reactions subject to further investigation (8).

## Mechanism of Action and Clinical use

**Figure 1:**
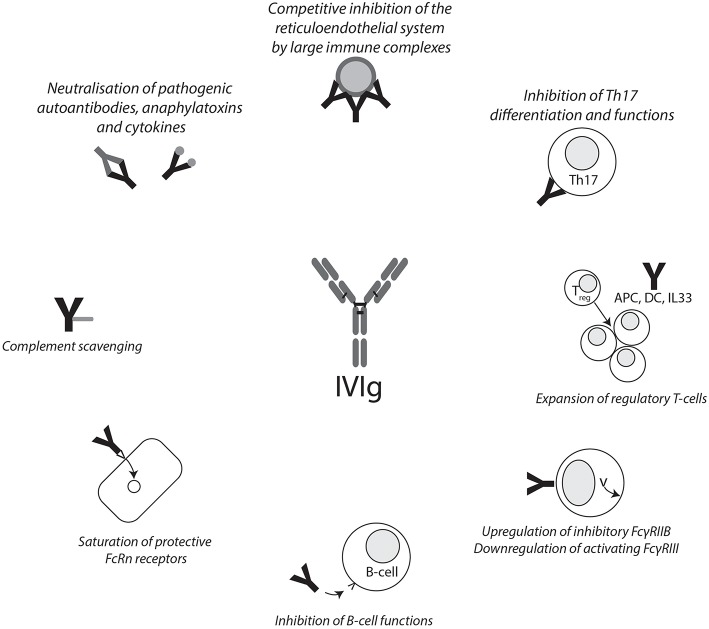
Summary of IVIg mechanisms of action. Graphical summary of reported mechanisms of action of IVIg. IVIg effects can be mediated through the F(ab)_2_ and Fc fragments. APC, antigen-presenting cell; DC, dendritic cell; FCγRIIB, Fcγ receptor IIB; FcRn, neonatal Fc receptor; IVIg, intravenous immunoglobulin.

The mechanism of action of IVIg in the treatment of autoimmune diseases is still a matter of debate. Generally speaking, one can discriminate between functions of the Fcγ region and the F(ab)_2_ region and their effects on a cellular level (Figure 1). These functions are not mutually exclusive, and given the heterogeneity of diseases IVIgs are used to treat, it is not unlikely that more than one pathway contributes to the beneficial effects. Indeed, there may be some truth to the statement that, after corticosteroids, IVIgs are the broadest immunomodulatory agent available (9).

Strong evidence for a prominent immunomodulatory function of the Fcγ fragment was reported as early as 1993, when Debre et al. (10) were able to show that transfusion of the purified Fcγ fragment is sufficient to successfully treat children with ITP. Indeed, IgG-binding Fcγ receptors were described on a variety of human cells including, among others, leucocytes and platelets. They are most effectively activated by cross-linking through binding of immune complexes. A leading hypothesis states that upregulation of the inhibitory Fcγ receptor IIB (FcγRIIB) is essential for the anti-inflammatory, immunomodulatory activity of IVIg. Supporting evidence for this hypothesis came from studies conducted by Samuelsson et al. (11), Siragam et al. (12), and Kaneko et al. (13), who could show that FcγRIIB expression is required for a response to IVIg infusion in murine models of ITP, arthritis, and nephritis, respectively. Through a series of high-impact publications, the group of Jeffrey Ravetch could subsequently demonstrate that the effects of IVIg on the Fcγ receptor repertoire were mediated not by binding of IVIg to a canonical Fcγ receptor but by a specific receptor for sialylated Fcγ fragments. Indeed, anti-inflammatory activity of IgG depends on specific sialylation of an N-linked glycan on the Fcγ region in mouse arthritis and nephritis models (14). Subsequent research demonstrated that a fully recombinant, appropriately sialylated Fcγ fragment is, in fact, sufficient to confer anti-inflammatory activity in a mouse arthritis model (15). Again using a mouse arthritis model, a specific C-type lectin, SIGN-R1, was later identified to be essential for the anti-inflammatory potential of appropriately sialylated IgG (16). Subsequently, the same group could demonstrate that humanized mice with macrophages and dendritic cells bearing the human orthologue of SIGN-R1, DC-SIGN, responded to IVIg treatment depending on DC-SIGN (17).

Another hypothesis, addressing mainly the context of ITP, states that large immune complexes are formed between IgG in IVIg and anti-D preparations and erythrocytes that inhibit macrophages competitively and via cytokine induction in the reticuloendothelial system, thereby preventing phagocytosis of platelets (18). While this hypothesis explains why anti-D IgG, sometimes given as an alternative to IVIg in ITP patients, is only effective in rhesus D–positive individuals (19), it is not easily compatible with reports that IVIg benefits in ITP were independent of the F(ab)_2_ fragment (see previous paragraph).

On a cellular and cytokine level, IVIg was reported to induce a Th2-type immune response with production of interleukin 33 (IL33) and IL4, leading to an increased expression of FcγRIIB on splenic macrophages in a murine arthritis model (17). It should be noted, however, that previous research has suggested that IL33 and IL4 are not crucial for the beneficial effects of IVIg in murine ITP models, and patients with ITP still respond to IVIg after splenectomy (20, 21). IVIg was furthermore reported to expand regulatory T-cells in a murine experimental autoimmune encephalitis model via interaction of IVIg with DC-SIGN on dendritic cells (22); to reduce expression of stimulatory cofactors, toll-like receptors, and additional activating pathways in B-cells *ex vivo* (23, 24); and to inhibit Th17 differentiation, amplification, and function *ex vivo* (25, 26).

Very recently, two recombinant IgG1 multimers were reported that confer either several (27) or only a subset (28) of IVIg effector functions and may offer new insights into the therapeutic role of IgG subunits in IVIg formulations.

Additional hypotheses on IVIg's mechanism of action will be discussed in the following paragraphs alongside the relevant dermatological conditions.

Table 1 summarizes indications for IVIg treatment in dermatology. Due to the rarity of some of these conditions, randomized clinical trials to support the use of IVIg are not generally available.

### Autoimmune Bullous Dermatoses

Autoimmune bullous dermatoses are a heterogeneous group of antibody-mediated autoimmune diseases, which manifest with bullae and erosions on the skin and mucous membranes. Prominent examples are pemphigus vulgaris and bullous pemphigoid. In pemphigus vulgaris, pathological antibodies against keratinocyte desmosomal proteins (desmoglein 3, desmoglein 1) cause intra-epidermal separation with the formation of blisters and erosions in the mouth, pharynx, and skin. Until the advent of steroid treatment, pemphigus vulgaris caused significant mortality due to malnutrition and superinfection. Even today, a subset of patients do not sufficiently respond to immunosuppressive and immunomodulatory treatment or succumb to fatal adverse effects. Bullous pemphigoid results from autoantibodies against hemidesmosomal proteins, in particular, the NC16A region of BP180. Therefore, and in contrast to pemphigus vulgaris, blister formation is sub-epidermal. The clinical course tends to be mild, and topical treatment may be sufficient. Other sub-epidermal autoimmune bullous dermatoses include epidermolysis bullosa acquisita, with anti-collagen VII autoantibodies and, consequently, the formation of deep blisters between the basal membrane and papillary dermis, and mucous membrane pemphigoid, which may result in scarring and, if affecting the eyes, loss of vision.

Treatment of recalcitrant pemphigus vulgaris and foliaceus and bullous pemphigoid with IVIg is supported by two randomized clinical trials (29, 30). Figure 1 documents the response of a patient to IVIg treatment. Usually, IVIg is used as a third-line adjunct treatment initially flanked by high-dose systemic corticosteroids and steroid-sparing immunosuppressants (5, 31). In analogy, IVIg is successfully used to treat other autoimmune bullous dermatoses including epidermolysis bullosa acquisita and mucous membrane pemphigoid (5). Once disease control is achieved, indicated by the healing of old erosions and no formation of new blisters, immunosuppressive treatment is slowly reduced and, finally, IVIg intervals may be extended to 6 weeks followed by cessation of IVIg treatment.

Regarding the mechanism of action, Li et al. (32) could demonstrate that response to IVIg treatment in murine bullous pemphigoid, pemphigus vulgaris, and pemphigus foliaceus models depended on the expression of the neonatal Fc receptor (FcRn), which regulates the half-life of serum IgG via pinocytosis and protection against lysosomal degradation. The authors put forward the hypothesis that IVIgs saturate FcRn receptors, resulting in accelerated degradation of pathogenic autoantibodies. Of note, FcRn-deficient mice developed only very mild symptoms of autoimmune bullous dermatoses, rendering the comparison with wild-type mice complex. The pathogenic relevance of FcRn in autoimmune bullous dermatoses was also demonstrated in murine experimental epidermolysis bullosa acquisita models, where FcRn deficiency protected from tissue injury [(33); for a review, see (34)]. Arguing against a prominent global role of FcRn saturation is the observation that FcRn deficiency does not preclude IVIg effects in mice with ITP (35). However, animal models of ITP usually focus on the initial phases of disease and treatment. Therefore, FcRn saturation may still contribute to the prolonged effects of IVIg treatment in autoimmune diseases. Other potential mechanisms that may contribute to IVIg's beneficial effects in autoimmune bullous dermatoses include anti-idiotypic antibodies against anti-BP180 IgG, which were demonstrated in IVIg and prevented BP180 degradation *ex vivo* (36), and a reduction of pro-inflammatory interleukin 6, which was recently demonstrated in IVIg-treated murine experimental bullous pemphigoid models (37).

### Dermatomyositis

Dermatomyositis is an antibody and complement-mediated microangiopathy characteristically affecting the proximal musculature of the extremities and sun-exposed areas of the skin (38). Apart from the musculature, systemic involvement mainly affects the heart and lungs. One-third of cases are paraneoplastic. Therefore, dermatomyositis is associated with considerable mortality. In contrast to adult dermatomyositis, pediatric dermatomyositis is usually not associated with malignancy.

The treatment of dermatomyositis with IVIg is supported by a randomized clinical trial (4). A recent guideline promotes the use of IVIg as a second-line therapy after failure of systemic high-dose corticosteroids, the mainstay of dermatomyositis treatment (5). This approach is of particular use when an associated malignancy is present to reduce the need for immunosuppressive medication. Alternative second-line treatments include steroid-sparing immunosuppressants like mycophenolate mofetil, azathioprine, and methotrexate (39). The immunosuppressive medications, starting with systemic corticosteroids, are slowly reduced once disease control is achieved. Finally, IVIg intervals may be extended to 6 weeks followed by cessation of IVIg treatment.

Basta and Dalakas (40) reported that IVIg treatment prevented the formation of the complement membrane attack complex in patients with dermatomyositis. Indeed, the Fcγ fraction of IgG was shown to bind C3b, thus opsonizing it for clearance by the reticuloendothelial system (41). Additionally, Basta et al. (42) could demonstrate that the F(ab)_2_ fraction of IgG binds and neutralizes the anaphylatoxins C3a and C5a using animal models of murine asthma and porcine C5a-induced cardiopulmonary distress. However, given the reported treatment effects of isolated Fcγ fractions, the F(ab)_2_ fraction may not contribute equally to effects of IVIg in other diseases including ITP [see above, e.g., (10)].

### Scleromyxedema

Scleromyxedema is histologically characterized by fibroblast activation, mucin deposition, and fibrosis and usually associated with paraproteinemia. The precise pathogenesis is, as of yet, unknown. Clinically, the depositions mainly affect the skin, where patients develop waxy papules and indurations that may lead to dermatogenic contractures. Systemic involvement of the peripheral and central nervous system, cardiopulmonary system, kidneys, joints, and/or muscles is observed in up to 30% of patients.

Given the unsatisfactory response and relevant associated morbidity of alternative treatments like systemic steroids, thalidomide, or melphalan, IVIg is recommended as a first-line treatment in severe scleromyxedema based on several case reports and series (5). Due to frequent relapses after cessation, long-term IVIg treatment is oftentimes necessary.

The pathogenic role of monoclonal IgG in scleromyxedema is unknown: while patient serum induced fibrosis in normal human fibroblasts, purified monoclonal patient IgG was ineffective (43). Hypotheses therefore include the blocking of an as of yet unknown circulating factor by IVIg (44). However, the mechanism of action is largely elusive and may involve various IVIg effects documented in other disease entities.

### Systemic Vasculitis

Vasculitides are a heterogeneous group of diseases that result from an inflammation of the arterial blood vessels and/or post-capillary venules. They are categorized, among other criteria, by the size of the affected blood vessels and single- or multi-organ involvement according to the Chapel Hill Consensus Conference criteria (45). The symptoms largely depend on the involved organ systems. In the skin, symptoms range from papules with hemorrhage (palpable purpura) to subcutaneous nodules and necrosis depending on the size and depth of the involved blood vessels. Regarding the pathogenesis, it is possible to discriminate those types of vasculitis that involve anti-neutrophilic cytoplasmic antibodies (ANCAs), leading to neutrophil activation and degranulation, from those that result from immune complex deposition.

A randomized controlled trial indicating beneficial effects of IVIg was conducted by Jayne et al. (46) in patients with granulomatosis with polyangiitis (GPA, formerly Wegener's granulomatosis), a potentially lethal form of ANCA-associated multi-organ vasculitis. Of note, some critics of the study have later objected to the short observation period of 3 months, and a recent Cochrane review found insufficient evidence to support the use of IVIg in GPA (47). Still, the EULAR advises using IVIg as an adjuvant treatment in GPA with moderate residual disease unresponsive to high-dose systemic corticosteroids, cyclophosphamide, and rituximab (48). Based on data on IVIg in ANCA-associated vasculitis and several case reports and series documenting beneficial effects of IVIg in a variety of vasculitides, the current European guideline promotes the use of IVIg in all severe systemic vasculitides in recalcitrant cases, if other therapeutic options are contraindicated, or if the disease takes a foudroyant course (5). Alongside acetylsalicylic acid, IVIg is considered a mainstay in the treatment of Kawasaki disease, as elaborated in the introductory part of this manuscript.

*In vitro* data indicate that anti-idiotypic antibodies contained in IVIg can bind and neutralize ANCA (49). Whether anti-idiotypic antibodies contribute to IVIg effects in the treatment of vasculitis *in vivo* is, however, uncertain. Of note, as previously detailed in this manuscript, the F(ab)_2_ fragment was not necessary to convey IVIg's beneficial effects in several other autoimmune diseases.

### Systemic Lupus Erythematosus

Systemic lupus erythematosus (SLE) is a multi-organ collagen vascular disease, which involves characteristic anti–double-stranded DNA (anti-dsDNA) autoantibodies in the serum. Extra-cutaneous symptoms include arthritis, lupus nephritis, esophagitis, enteropathy, hepatitis, pneumonitis, interstitial lung disease, pulmonary hypertension, pericarditis, endocarditis, encephalitis, peripheral neuropathies, hematologic abnormalities, and thromboembolic disease. The skin is usually affected in a UV-dependent fashion. Individual lesions can range from erythematous macules on both cheeks (butterfly rash) to scarring erythematous, scaly plaques, and panniculitis.

The treatment of SLE depends on the involved organ systems and includes a variety of immunosuppressive agents [for a detailed review, cf. (50)]. Two randomized controlled trials reported beneficial effects of IVIg in SLE in the context of pregnancy in patients with recurrent abortions and in the treatment of lupus nephritis (51, 52). According to the European guideline, IVIg should be considered as a treatment option in all cases of severe lupus erythematosus (5). In particular, IVIg may be beneficial in the treatment of pregnant women, SLE-associated ITP, stable lupus nephritis, and neuropsychiatric disease (53).

Interestingly, affinity-purified anti-idiotypic antibodies against anti-dsDNA were effective in the treatment of an experimental murine SLE model (54). However, several of the previously detailed mechanisms can be hypothesized to contribute to the beneficial effects of IVIg in SLE [for a review, see (55)].

## Outlook

IVIg is successfully used to treat an expanding range of autoimmune diseases. While strong evidence points toward a prominent role of the Fcγ fragment to convey the beneficial effects of IVIg treatment, there may be more than one mechanism of action, and their precise role may vary depending on the disease in focus. It is, however, tempting to speculate that only a fraction of the components contained in IVIg formulations, e.g., specifically sialylated Fcγ fragments, are responsible for their clinical effects and that more refined preparations may make the infusions more accessible and even safer in the future.

## Author Contributions

JH wrote the first draft of the manuscript. JH and AE revised the work for important intellectual content. JH and AE read and approved the submitted version.

### Conflict of Interest Statement

The authors declare that the research was conducted in the absence of any commercial or financial relationships that could be construed as a potential conflict of interest.
